# Alienation and Abstract Labor-Time: Marx and the Romantic Critique

**DOI:** 10.1177/00368237251383447

**Published:** 2026-02-13

**Authors:** Sean K. Isaacs

**Keywords:** temporality, anti-colonial, Marxism, romanticism, alienation

## Abstract

Capitalist commodity production subordinates concrete and distinct forms of creative activity to the quantifiable and homogeneous category of abstract labor. In order for commodities to be exchanged on the market, products of specific types of human labor must be equalized as measurable units of abstract labor-time. Capital’s logic of value separates us from the essential human capacity to labor, turning human activity into a means of obtaining a wage for workers and producing surplus-value for capitalists. Marx’s theory of alienation shows that, under capitalism, workers have been alienated from their species-being, or their ability to fulfill biological and social needs through conscious activity in and on the world. This process reduces qualitatively distinct forms of human activity to quantifiable units of abstract labor-time. The subordination of concrete labor to abstract labor-time alienates workers from their essential human capacity to consciously and creatively act in and on the world—their ability, that is, to engage in unalienated labor—while also subsuming non-capitalist forms of time to the logic of value production. Anti-colonial resistance to the subordination of all forms of life to the logic of value represents a concrete example of the struggle against alienation in practice. By viewing elements of the past as an ongoing site of contestation, Romantic Marxism seeks to combine a living tradition of life and resistance with working-class organizing in the present, as part of a movement toward a qualitatively different communist society in the future. Integrating the Romantic view of the past with Marx’s critique of alienation expands the utopian vision of communist freedom, while remaining grounded in, and emerging from, the material conditions of the present.

## Introduction

Capitalist commodity production subordinates concrete and distinct forms of creative activity to the quantifiable and homogeneous category of abstract labor. In order for commodities to be exchanged on the market, products of specific types of human labor must be equalized as measurable units of abstract labor-time. Capital’s logic of value separates us from the essential human capacity to labor, turning human activity into a means of obtaining a wage for workers and producing surplus-value for capitalists. Marx’s theory of alienation shows that, under capitalism, workers have been alienated from their species-being, or their ability to fulfill biological and social needs through conscious activity in and on the world. While alienation is grounded in the labor process, it extends to all aspects of social life. Therefore, in order to overcome capitalism, workers must overcome alienation; they must, in other words, regain control over the labor process, over the time in which they exert their labor-power, thereby allowing for the complete reorganization of social relations. The struggle against alienation is only possible because capitalism continues to rely on concrete and specific forms of labor to produce commodities, which must satisfy a real human need. Because concrete forms of labor and life remain necessary for capital’s reproduction, the potential to recover elements of non-capitalist ways of living represents part of the struggle against alienation. Anti-colonial resistance to the subordination of all forms of life to the logic of value represents a concrete example of the struggle against alienation in practice. By viewing elements of the past as an ongoing site of contestation, Romantic Marxism seeks to combine a living tradition of life and resistance with working-class organizing in the present, as part of a movement toward a qualitatively different communist society in the future. Integrating the Romantic view of the past with Marx’s critique of alienation expands the utopian vision of communist freedom, while remaining grounded in, and emerging from, the material conditions of the present.

The notion that a theoretical break can be found in Marx’s work, between his early “humanist” tendencies and his later “scientific” developments, has been a subject of Marxist debate for decades. Most famously argued by Louis Althusser (2005), the contention that 1845 was the point when Marx rejected his ethical and humanist critique of capitalism is undergoing a revival today (see Mau 2023). For Søren Mau, Marx’s early critique of alienation is plagued by both humanist and romantic tendencies, “humanist in the sense that the concept of the essence of the human being occupies a central role as the basis of critique, and romantic because it is based on an idea of an original, lost, and natural unity which ought to be restored” (82). This leads to a political project seeking a return to an imagined unity with an unalienated human nature. However, Marx’s theory of alienation is essential to understanding his conception of communist freedom, which views human emancipation as self-determined, unalienated activity. An authentic version of communism is only possible by regaining control over the labor process and reclaiming the time in which we labor, by recovering what Marx calls our species-being. By locating labor as the basis of Marx’s theory of alienation in the *Economic and Philosophical Manuscripts of 1844*, I argue that his concept of commodity fetishism developed in *Capital*, where concrete human labor appears as abstract, value-producing labor, builds upon his earlier work on alienation.

This paper sits within Marxism’s “warm current” (Bloch 1986), that which does not separate Marx’s ethical critique from his theory of capitalism, and draws from Marx as well as the utopian and Romantic traditions that influenced him. Löwy and Sayre (2001) have noted the connection between Marx and Romanticism, specifically the total critique of the quantification, mechanization, and dehumanization of human life that has occurred under modern capitalist civilization. Their broad definition of Romanticism, which I follow here, understands it to be a critique of modern industrial capitalism which draws from the pre-capitalist past. While the Romantic tradition is heterogeneous, in that it can take both revolutionary and reactionary forms, the revolutionary current expresses a tragic-utopian dialectic in its critique of modernity, both a nostalgic lament for what has been lost and a sense of hope for what might still be realized. Romanticism so conceived, what Nikolas Kompridis (2015) calls “political romanticism” (1), both critiques and affirms modernity. It represents a living tradition desirous of qualitative, transformative, and material change through the formation of new forms of organization and social life while drawing selectively from the past. In this sense, it is “a modern critique of modernity” (Löwy and Sayre 2001, 21), emerging from the experience of capitalism while criticizing it for failing to live up to its promise of universal freedom.

The tension between tragedy and utopia can be found in Gothic literature of the nineteenth century, tales of the violent and horrific erasure of life that has occurred as capitalist relations have spread across the globe (McNally 2011). Vampire stories emerged from the conditions of an industrializing Britain, conjuring images of the working masses drained of their creative life-force by the dehumanizing power of capital. However, as David McNally argues, the tragic dimension is dialectically related to its possible overcoming, for at the same time that capital directs living labor toward the production of surplus-value, it also brings together a class of workers that could overcome it. This “monstrous dialectic” (253) is, for McNally, best expressed in Mary Shelley’s 1818 Gothic novel *Frankenstein*, where Frankenstein’s Creature represents both a monstrous, alienated creation of modernity and a conscious, living being seeking to reclaim the universal right to human life.

Marx’s theory of alienation is itself a product of his humanist and romantic influences. For Romantic thinkers like Heinrich Heine, universal human freedom cannot be achieved either under modern bourgeois society or through a return to the past, because prior forms of life were themselves based upon oppressive and exploitative relations (Löwy and Sayre 2001, 74–5). Thus, while the spread of modern capitalism has occurred through a process of dispossession, expropriation, and colonization, damaging pre-existing relations of mutuality and reciprocation amongst human beings as well as between humans and nature, it cannot be overcome through a wholesale return to the past. For Marx, 1978b, alienation can only be overcome when individuals realize their “species-being,” reclaiming their “social power” (46) to labor as their own human essence. Here we can see the influence of Romanticism, the need to transcend alienation and reunite human beings with their essential ability to act in and on the world. But because alienation is, for Marx (1988), historically specific to capitalism, its overcoming must occur in relation to the particular level of development of human society.

Though Marx was certainly not blind to the progressive features of capitalism, and, at times, celebrated it for achieving a level of development that had not been reached before, his work nonetheless drew extensively from the Romantic tradition and maintained a utopian vision of a future communist society. Marx’s early work was heavily influenced by Romantic literature, and this persisted throughout his life. *Capital* contains multiple references to Gothic imagery, including capital’s “vampire-like” (Marx 1990, 342) character, as well as to Charles Dickens’—one of Marx’s favorite authors—*Oliver Twist* (569). *The Communist Manifesto* notes that the works of Utopian Socialists “are full of the most valuable materials for the enlightenment of the working class” (Marx and Engels 2002, 255) and Marx’s later writings expressed an admiration for pre-capitalist communal traditions in North America, India, and Ireland, amongst others (Anderson 2025).

These Romantic threads allowed Marx to acknowledge the fact that capitalism has allowed for a level of human development not seen in prior stages of history while remaining critical of the way that it has produced new and more brutal forms of exploitation, violence, and alienation. In this sense, the Romantic influence on Marx can be directly tied to his evolving views on non-Western societies and anti-colonial resistance. His work, particularly later in life, often expressed admiration for past, more human ways of relating to each other and the natural world, including North American Indigenous societies and communal traditions in Ireland and Russia (Anderson 2016; 2025; Rosemont 2009). As a result, he came to view peripheral countries, like India, Ireland, and Russia, as central to the possibility of world communism, if their anti-colonial struggles and communal traditions could be combined with the broader proletarian movement in Western Europe (Anderson 2016, 2025). Rather than viewing anti-colonial movements as isolated or distinct, Marx viewed them as particular expressions of the struggle for *universal* freedom.

In what follows, I begin by tracing Marx’s theory of alienation from his early works to *Capital*. Drawing especially on the *Economic and Philosophical Manuscripts of 1844*, the first section will focus on the relationship between labor and needs in human society as the basis of Marx’s theory of alienation. The second part follows the concept of alienation through *Capital*, connecting it with Marx’s theory of commodity fetishism. The commodity fetish does not replace alienation but represents a specific development of the concept. By examining the contradictory relationship between abstract and concrete labor, Marx shows how specific forms of human creative activity are quantified and made exchangeable through the category of abstract labor, reducing labor-power into a commodity to be sold for wages on the market and a means of producing surplus-value for capitalists. The third part considers the political consequences of this way of conceiving alienation. The reduction of concrete forms of human activity to abstract labor has a temporal dimension, where multiple and diverse concrete times are subsumed to the logic of value and made quantifiable and measurable as abstract labor-time. The struggle to overcome alienation, therefore, is a struggle to reclaim the concrete times of labor and life. The fourth and final part highlights the importance of the Romantic critique in Marx’s work. Recovering concrete forms of labor and time is only possible because elements of the past persist as a part of capitalism’s uneven totality. Marx himself saw anti-colonial struggle, including the persistence of communal forms of living, as the potential basis for a different future, if they could be combined with working-class movements in the present. Romantic Marxism views the selective recovery of elements of the past as an essential part of the struggle against alienation in the present, an effort to materially realize Marx’s utopian vision and re-establish a more human connection with ourselves, each other, and the world.

## Romantic Influence in the Young Marx

The concept of alienation predates Marx, beginning with the Judeo-Christian notion of the “Fall from Grace” and the messianic project of reuniting humanity with the kingdom of God (Mészáros 2005). This view was secularized through the notion of “saleability” (33) or the freedom to sell, or alienate, anything, including human labor-power. This resulted in what Thomas Carlyle calls “mammonism” (Löwy and Sayre 2001, 35), or the worship of Money. This process of commodification required divorcing human activity from the individual, transforming labor-power into a thing to be sold on the market. Jean-Jacques Rousseau was one of the harshest critics of alienation prior to Marx and denounced the separation of the individual from freedom, from sovereignty, and from nature (Mészáros 2005). Rousseau linked these forms of alienation to the rise of market society. But while he was critical of existing institutions, private property remained at the basis of Rousseau’s theorization of civil society, making him unwilling and incapable of questioning the legal forms that upheld property rights, thereby limiting his critique of alienation to an ideological one.

In his early works, Marx was primarily focused on the modern state and the division between the public citizen and the private individual (Kouvelakis 2003; Leipold 2024; Leopold 2007; Löwy 2005; Mészáros 2005). However, he consistently placed capitalist society and private property at the center of his analysis. In the Introduction to *A Contribution to the Critique of Hegel’s Philosophy of Right*, Marx (1978a) shows the centrality of the proletariat’s demands for “the negation of private property” (65) to a political project aimed at “complete emancipation” (64). While alienation in the work of previous thinkers like Rousseau was purely ideological, based on the separation of the individual from an abstract human nature, Marx grounded his theory in the concrete analysis of capitalist social relations.

In the *Manuscripts of 1844*, Marx (1988) maintains that the human being is a “natural being” (154). This is “subjectively” true, in the sense that humans are able to enact their “essential powers” on the natural world and also “objectively” true because humans, like the rest of nature, can only express these powers by interacting with an objective world outside of themselves (Soper 1986, 35). In Marx’s (1988) words:

As a natural being and as a living natural being he is on the one hand furnished with *natural powers of life*—he is an *active* natural being. These forces exist in him as tendencies and abilities—as *impulses*. On the other hand, as a natural, corporeal, sensuous, objective being he is a *suffering*, conditioned and limited creature, like animals and plants. That is to say, the *objects* of his impulses exist outside him, as *objects* independent of him; yet these objects are *objects* of his *need*—essential *objects*, indispensable to the manifestation and confirmation of his essential powers. (154.)

But alienation for Marx occurs under historically specific capitalist social relations, and this is what distinguishes Marx’s theory of alienation from the more abstract and idealist views that preceded him. While human beings must “objectify” their powers in all societies, in that they act on an objective natural world, it is only in capitalist society that these powers appear to exist outside the individual. Alienation is therefore a historical process that occurs as humans are separated from their ability to fulfill their biological and social needs.

But the relationship between human needs and the necessity of fulfilling them is also, in part, transhistorical. There are certain objective, biological needs that exist throughout human history—hunger, for instance—and it is only possible for human beings to fulfill these needs through their inherent ability to act in, on, and with an objective nature outside of themselves. In other words, objective biological needs are the most basic limit to the enactment of our labor-power (Geras 1994). But humans are not only natural beings; they are also “*human* natural” (Marx 1988, 155, all emphasis in original unless otherwise noted) beings, which is to say that, unlike other animals, they are conscious of the historical and social process of their own development through their interaction with the natural world. As human labor develops, so does the ability of humans to appropriate nature within the confines of objective limits, both natural and historically specific. This increases the complexity and ability of humanity’s powers while also expanding and diversifying their social needs. Consciousness of historical and social processes makes up a part of human nature, which is neither entirely determined by biological limits nor entirely outside of them (Soper 1986). David Camfield (2014) makes a similar point when he argues that while there is a biological dimension to human nature, biology does not determine the entire range of what is possible. There are “emergent properties” (292), including gender, sexuality, and race, that are not derived from biological nature. From this perspective, human nature is neither merely the idealized and determining element of the form human activity takes in all societies, nor is it entirely shaped by the particular social relations of any given historical period. In this way, it “distinguishes itself both from idealism and materialism, constituting at the same time the unifying truth of both” (Marx 1988, 154).

But what is Marx referring to specifically, what are these “powers” that have been alienated from us? In the *Manuscripts of 1844*, Marx (1988) identifies labor, our ability to consciously and creatively act on the world, as the essence that has been alienated by capitalist society. He highlights four ways in which humans have been alienated under capitalism; from the natural world, in that the products of labor appear to be external to them; from themselves, in that their own labor appears to confront them as an outside force; from their species-being, in that human conscious, creative activity (labor) becomes merely a means to existence rather than the end goal of life itself; and from other people, in that once one’s own labor is alienated, the activity of others likewise seems external and individualized. Each of these forms of alienation are related to the other, but it is the notion of species-being that represents our human essence:

It is *its life-activity*. Man makes his life-activity itself the object of his will and of his consciousness. He has conscious life-activity. It is not a determination with which he directly merges. Conscious life-activity directly distinguishes man from animal life activity. It is just because of this that he is a species being. Or it is only because he is a species being that he is a Conscious Being, that is, that his own life is an object for him. Only because of that is his activity free activity. Estranged labor reverses this relationship, so that it is just because man is a conscious being that he makes his life activity, his *essential* being, a mere means to his *existence*. (76)

Here, Marx shows that capitalism has alienated workers from their ability to consciously act on the world. Rather than appropriating and transforming the world as an expression of their potential for creative work, labor under capitalism becomes a mere means of survival, something external to them: “the more the worker *appropriates* the external world, sensuous nature, the more he deprives himself of *means of life* in the double respect: first, that the sensuous external world more and more ceases to be an object belonging to his labor—to be his labor’s *means of life*; and secondly, that it more and more ceases to be *means of life* in the immediate sense, means for the physical subsistence of the worker” (72). Under capitalism, labor, or the specific way in which workers appropriate the world and objectify their essential powers, becomes an expression of alienation. Rather than a process that fulfills biological and social needs and enriches the individual and their community, labor appears as a something outside us, “a mere means to existence.”

Labor’s alienation, the transformation of our essential human capacity to create into a means of survival alienates *all* social relations under capitalism, distorting qualitatively distinct forms of human life and reducing them to homogeneous, quantifiable money relations (Sears 2025). In the *Manuscripts of 1844*, Marx (1988) argues that “[s]ince money as the existing and active concept of value, confounds and exchanges all things, it is the general *confounding* and *compounding* of all things—the world upside-down—the confounding and compounding of all natural human qualities” (140). It is for this reason that Marx’s critique of alienation takes aim at the totality of capitalist social relations. This total critique retains Marx’s romantic influence and mourns the quantification, mechanization, and dehumanization of the world that has occurred under modern capitalism. However, unlike romantic anti-capitalists, Marx maintains a dialectical critique of capitalism, allowing him to recognize that while capitalism has created new needs and expanded our capacity to fulfill our needs, it has also alienated us from these more developed powers (Wills 2024).

## Alienation in *Capital*

Some, like Althusser (2005), have argued that Marx moved beyond the concept of alienation by the time he wrote *Capital*. However, this disregards the evolutionary trajectory in Marx’s work, from alienation and species-being in the *Manuscripts of 1844*, to commodity fetishism in *Capital*. Indeed, “it was Marx’s habit to return to his earlier notebooks in drafting his later works” (Ollman 1977, xv) and *Capital* retains the ethical and Romantic essence of his earlier work, even if it discards some of the terminology (Wills 2024). Contrary to Søren Mau’s (2023) assertion that Marx “changed his mind” (83) after 1845, Volume I of *Capital* makes many references to the separation of workers from their labor and its products that occurs under capitalism that bear a remarkable resemblance to the *Manuscripts* (Wills 2024). For instance, Marx (1990) describes the quantification of human labor that occurs under capitalist commodity production, where “[d]efinite quantities of product, quantities which are determined by experience, now represent nothing but definite quantities of labor, definite masses of crystalized labor-time. They are now simply the material shape taken by a given number of hours or days of social labor” (297). By subordinating concrete human experience to “quantities of labor,” capital’s logic of value erases the particular qualities of distinct forms of human labor, while labor’s products are reduced to measurable units of abstract labor-time. As a result, labor ceases to be an expression of human creative activity. Instead of contributing to human flourishing, the development of productive relations in capitalist society “distort[s] the worker into a fragment of a man, they degrade him to the level of an appendage of a machine, they destroy the actual content of labor by turning it into a torment; *they alienate from him the intellectual potentialities of the labor process* in the same proportion as science is incorporated in it as an independent power” (799, emphasis mine). Here, capitalist labor separates humans from their essential powers, from their capacity to consciously and creatively act on the world, making labor appear as an abstract force, “an independent power” outside of the worker. While capitalism has allowed for the development of human productive capacities to levels that were previously not possible, these powers have been directed toward the growth of capital, rather than the enrichment of the lives of workers (Wills 2024). To use the language of the *Manuscripts*, this process has alienated humans from their species-being, from their capacity to enact their social powers in and on the world.

This throughline from the *Manuscripts of 1844* to *Capital* can be found in the category of labor and its relationship to needs. In the *Manuscripts*, Marx identifies labor as humanity’s essence. In *Capital*, he reveals the substance of capitalist value to be abstract labor, measured in socially necessary labor-time, or the average time a society takes to produce something under normal conditions, including the level of productivity of society and the level of skill of its workers (Marx 1990). But a commodity is not only a value; it must also be a use-value, it must satisfy a real social need. To do so, it must be produced by concrete forms of human activity, the specific act of making a table, for instance. However, because capitalist production occurs for the market rather than in response to human needs, human labor is separated from those who do the producing. By directing production toward exchange on the market, human development has been alienated from the productive powers of society (Wills 2024). Labor becomes a means to obtain a wage for the worker and produce surplus-value for the capitalist, rather than an act of self-realization allowing for the satisfaction of human needs (Brixel 2024).

In order to become exchangeable, value must be expressed as exchange-value, it must be quantified as abstract labor-time. Thus, its particular useful quality, its ability to satisfy human needs, is abstracted away:

It is no longer a table, a house, a piece of yarn or any other useful thing. All its sensuous characteristics are extinguished. Nor is it any longer the product of the labor of the joiner, the mason or the spinner, or of any other particular kind of productive labor. With the disappearance of the useful character of the products of labor, the useful character of the kinds of labor embodied in them also disappears; this in turn entails the disappearance of the different concrete forms of labor. (Marx 1990, 128)

The “disappearance” of these particular forms of labor refers to the quantification of what are in actuality qualitatively distinct types of human activity. This occurs through the capitalist category of exchange-value and the subordination of living labor to dead labor (Wills 2024). The specific form of labor, that which makes it the labor of the joiner, the mason, or the spinner, is erased by way of capitalist abstraction, transforming human labor into a means of producing value, which appears to be the result of the movement of capital itself. Labor under capitalism is no longer an end in itself, the enactment of our species-being, the fulfillment of social needs, and the collective enjoyment of the products of our free, conscious, creative activity. It is alienated labor, a means to an end, directed only toward the individual, selfish need to realize value on the capitalist market (Chitty 1993). As Pascal Brixel (2024) has argued, this makes the content of labor insignificant to the worker, separating their creative activity from their own life. At the same time that capitalism has expanded the potential for human labor and created new social needs, it has also directed labor away from human fulfillment and toward the production of surplus-value.

This contradictory relationship, between concrete human activity and abstract labor is what Marx (1990) calls “the dual character of labor” (131). Both use-value and value find their origin in labor, but while the production of use-values requires specific types of human activity, the production of value abstracts from these qualitatively distinct forms of labor. However, while use-value has been subordinated to the law of value, Marx insists that concrete human labor, that which produces use-values, persists under capitalism. Therefore, while a commodity only has value if it is also a use-value, a product of labor can be useful without being a commodity, without being productive of value. This occurs whenever someone directly fulfills a social need through their own labor. The dual character of labor refers to the tension between the essentially human capacity to act in and on the world that exists in all societies on the one hand, and the specific form that labor takes under capitalism on the other. This is the tragic-utopian dialectic that an attentiveness to Marx’s romantic and humanist influences helps reveal. While capitalism tends to reduce all types of human activity to the abstract and measurable form of value-producing labor, it does not—indeed, it cannot—fully eliminate the transhistorical human element of labor, that which fulfills human needs.

### Alienation and the Fetishism of Commodities

Understanding capitalist labor in this way, as concrete human activity on the one hand and as abstract, value-producing labor on the other, allows us to trace the continuity from the *Manuscripts of 1844*, where Marx explicitly theorizes alienation, to *Capital*, where he develops the concept of commodity fetishism. While alienation, for Marx, indicates the way labor begins to appear as a force outside the worker, commodity fetishism describes capitalist society as one where social relations between people are expressed and viewed as relations between things (Marx 1990). Both Marx and Engels were influenced by Carlyle’s criticism of mammonism, or the worship of money in English society (Löwy and Sayre 2001, 90). Under capitalism, what are actually social qualities, relations between people, appear as intrinsic and natural qualities of commodities, of the products of social labor. Furthermore, commodity exchange also “seems to be the result of the movement of money” (Marx 1990, 212). This originates in the particular character of labor under capitalism and obscures the actual relations that exist between people. As Marx, puts it, “the commodity reflects the social characteristics of men’s own labor as objective characteristics of the products of labor themselves, as the socio-natural properties of these things” (164–65). While some, including Mau (2023), insist that the concept of the commodity fetish should be interpreted merely as an “*ideological* form” (193), such an approach fails to see that Marx’s theory of fetishism is central to his theory of value. Rather than the commodity fetish being simply an ideological appearance under capitalism—a mere illusion in which social relations between people are masked by relations between things—social relations *actually are* mediated and expressed through things (Rubin 2019). This is because, under capitalism, the people who produce goods for society do so not in response to social needs but as isolated units producing for the market, only brought together by the need to exchange their products. In other words, they are producing under conditions of alienated labor.

Rather than being directed toward the reproduction of human beings, toward the fulfillment of social needs, capitalist production is directed toward the production of surplus-value, which is only realizable through exchange on the market. This not only masks the relations between people which allow for commodity exchange to happen at all, it also creates new, alienated relations, where social production can only occur if it is mediated by commodity exchange on the market (Rubin 2019). In a non-capitalist society, where those who produce social goods are not separated from the means of production or from one another, production could respond directly to the needs of society. Labor would be social in that tasks would be divided amongst the members of the community based on the biological and social needs of that society and the existing ability to fulfill them, the level of development of that society’s productive relations (and, indeed, the level of development of productive relations at the global scale). But in a capitalist society, production does not respond to social needs. It cannot, because production is individuated by the market, meaning that no individual producer can know how much of any product is needed, nor how much is being produced in total. Producers are only brought together by the market, through the exchange of their products. In other word, they only relate to each other through things. Production under capitalism is directed by the logic of value and labor is organized according to the exchange of things on the market by isolated producers. But, as Marx (1990) insists, exchange relations are only a surface level appearance. Capital’s logic can only be revealed by understanding the way that labor is carried out, and this is why Marx leaves the “noisy sphere” of exchange and enters the “hidden abode of production” (279).

In this sense, fetishized relations are, indeed, an “appearance,” but not just in the ideological sense. They are the surface level, but all-too-real way that people relate to one another in capitalist society. But social relations under capitalism are not reducible to this surface appearance. As Marx (1990) shows, classical political economists took these fetishized relations to be natural, viewing the form that social relations take under capitalism as essential to human society throughout history. Marx’s project was to get behind the commodity fetish, to reveal the essence of capitalist value production, the productive relations between human beings. It is precisely these latter relations that have been alienated by capital. Under capitalism, concrete human labor is abstracted and quantified. It is measured as abstract labor-time, making the actual relations between workers appear to be the result of commodity circulation on the market. Rather than viewing commodities as the product of their own creative activity, workers view their labor as having a power over them. In order to gain access to the products of their work, they must continue to sell their labor-power for a wage. Thus, the subsumption of qualitatively distinct forms of human activity to abstract labor represents a separation of the worker from both the product of their labor and their labor itself: “To the producers, therefore, the social relations between their private labors appear *as what they are*, that is, they do not appear as direct social relations between persons in their work, but rather as material relations between persons and social relations between things” (165–66, my emphasis). Fetishized relations, therefore, are both real and illusory. The subsumption of concrete human activity to abstract labor that Marx describes in *Capital* is an extension of his theory of alienation. As Lucio Colletti (1972) states, “‘[a]bstract labor’, in short, is *alienated* labor, labor separated or estranged with respect to man himself” (84).

## Alienation and Time

### Abstract Labor and Abstract Time

Labor under capitalism requires the quantification of human activity, the reduction of qualitatively different forms of labor to socially necessary labor-time (Marx 1990).^1^ Because capitalist production must be mediated by the market, the satisfaction of human needs can only occur through exchange. The logic of value appears as the driving force of production, as the reason that human labor occurs at all. This “appearance” is the expression of real capitalist relations, of the fact that production *does* only occur for the market under capitalism.

Abstract labor, then, is not simply the reduction of specific types of labor to the general form of labor-power. It is the specifically capitalist way in which concrete labor can *only* be realized by way of its reduction to abstract labor-time, by exchanging the commodity labor-power for wages on the market. Thus, “abstract labor arises from the alienation of individual labor” (Rubin 2019, 109), from the specifically capitalist way that human activity is quantified for sale on the market. Capitalist abstraction erases qualitatively distinct forms of labor and separates them from workers. Labor-power is alienated from the worker, becoming a thing to be sold. Through this process of alienation, labor comes to be seen as a power outside of the worker, one which dominates them through the power of abstract labor-time.

Because capitalist workers are not slaves, they only sell their labor-power for a specific period of time (Marx 1990). For that time, the worker’s labor-power belongs to the capitalist and can be used to not only reproduce the capital that is paid out in wages—necessary labor-time—but to produce more value. Surplus-value is produced during what Marx calls surplus labor-time, so it is in the capitalist’s interest to maximize the time the worker spends producing for them, since the longer a worker labors (at the same level of intensity) beyond the time it takes to reproduce the value of their wages, the more surplus-value they produce. By alienating workers from their labor, by compelling them to sell their labor-power for a wage, abstract labor-time comes to dominate the lives of workers, structuring their days even when they are not at work. While concrete labor occurs according to the specific rhythm of the task at hand and can therefore be expressed in a multitude of ways, capital seeks to extract as much surplus labor from workers as possible (Martineau 2016). Capitalist production, therefore, strives to speed up the production process as much as possible, regulating the worker’s activity so as to increase the amount of surplus-value being produced. This requires a form of time discipline that controls workers’ activity and maximizes the time they spend working for capital. It requires, in other words, the subsumption of the multiple and differential rhythms of concrete human activity to the time of capitalist production, to abstract labor-time.

### Alienation and the Struggle Over Time

The alienation of abstract labor is, therefore, also the alienation of the concrete times in which labor occurs. As Jonathan Martineau (2016) shows, in order for capital to make the exchange of qualitatively different forms of human activity possible, qualitatively distinct forms of time must be made into quantifiable and homogeneous units of abstract labor-time. This was accomplished in part by the subjection of daily life to the time of the clock and the institutionalization and coordination of capitalist relations of production globally through the spread of World Standard Time (50). The subordination of the multiple and diverse concrete times of life and labor to abstract labor-time is what Martineau calls “a specific form of temporal alienation” (133). Not only is a worker’s capacity to labor alienated from them, but the very time of their life is transformed into a means to obtain wages in order to survive. The multiple and diverse rhythms of human activity are reduced to “empty and homogeneous time-units” (117), subsumed to the logic of value production. Drawing from Gothic imagery, Marx (1990) describes this as the triumph of “dead labor which, vampire-like, lives only by sucking living labor, and lives the more, the more labor it sucks” (342). Capitalist time, that which reduces living human rhythms to the time of value production, is likewise dead or “empty” time, the time in which capital, in its “vampire-like” manner, extracts surplus-value from living labor.

Abstract labor separates the worker from their capacity to enact their essential powers and subordinates them to the abstract time of capital, thereby limiting the time they have for themselves, for the enrichment of their own lives and the lives of their communities. It alienates humans from their species-being by directing their labor toward the production of value rather than human flourishing (Colletti 1972). By reducing human beings to mere holders of the commodity labor-power, capital sees workers as “nothing other than labor-power for the duration of his whole life” (Marx 1990, 375). A worker’s time becomes merely the abstract time of value production, the time of capital. This leads to workers only feeling human in their free time outside of work, while they feel the most unfree at work. But even “free time” is not the worker’s own. It is limited by the working day and is often spent recovering from work, performing necessary reproductive tasks, or escaping from the reality of going to work the next day through alcohol, drugs, or television. As Marx (1988) states: “As a result, therefore, man (the worker) no longer feels himself to be freely active in any but his animal functions—eating, drinking, procreating, or at most in his dwelling and in dressing-up, etc.; and in his human functions he no longer feels himself to be anything but an animal. What is animal becomes human and what is human becomes animal” (74).

But while capitalism seeks to subsume all concrete times to the abstract time of value production, this process remains incomplete. Multiple and differential human rhythms persist despite the hegemony of abstract labor-time. This is rooted in the dual character of labor, the tension between concrete and abstract labor. While abstract labor under capitalism must be expressed as homogeneous and measurable units of labor-time, the production of value still requires specific, qualitatively distinct forms of human creative activity occurring in concrete times, according to the specific rhythms of labor (Martineau 2016). It is for this reason that Marx (1990) sees the struggle over the working day, the struggle over time, as so important. It is the way that workers attempt to assert control over the time of their lives, it is a struggle between living and dead times. This occurs at the workplace, where workers often try to maintain and impose their own rhythms of work, both informally during the course of their time at work and formally, by setting legal limits to the working day. The struggle over time also takes place outside the point of production in the sphere of social reproduction, where social reproductive workers seek to regain control over their labor by insisting that it be directed toward human well-being, rather than the reproduction of capital (Ferguson 2023; Martineau 2016).

There is a colonial dimension to the subordination of human rhythms to the time of capital. Silvia Federici (2014) highlights the ways that violent processes of colonization have been central to the subordination of pre-capitalist forms of life. The spread of capitalist social relations requires the dehumanization of colonized subjects and their forcible separation from the ways of living that sustain them. This process requires the subordination of concrete and differential rhythms of non-capitalist ways of life to capitalist production time (Martineau 2016). The imposition of World Standard Time occurred, in part, through forms of colonialism and imperialism, attempts to subordinate pre-colonial forms of life to the logic of value, as occurred during the British colonization of Australia (155–9). But while capitalism has spread to all corners of the globe, indigenous resistance to the colonial imposition of abstract labor-time persists, both through anti-colonial struggle and in communal ways of life. These forms of resistance seek to defend “not-just-capitalist” (Kipfer 2018, 486) lifeways and alternative conceptions of time. Indeed, Aboriginal resistance to British colonization in Australia sought to maintain cyclical and seasonal understandings of time, and these struggles continue to this day (Martineau 2016, 156–9). The framework of Romantic Marxism allows us to understand anti-colonial struggles as attempts to draw from living indigenous traditions, not as isolated efforts to return to an idealized past, but as part of a broader movement intent on overcoming capitalism and creating a qualitatively different future.

## The Romantic Critique

Some have criticized any appeal to the past for its humanist and romantic tendencies. For example, Mau (2023) argues that “the political project which follows from such a critique necessarily takes the form of *the reconstitution of a natural order*, that is, the emancipation of human nature or the abolition of capitalism in order for humans to *become what they really are* underneath their alienated existence” (82). Here, human nature is taken to mean a pure and untouched human essence that has been defiled by capitalist relations. Mau argues that the concept of species-being is merely a type of *consciousness*, while Marx’s more developed economic theory is based in *production*. However, even in the *Manuscripts of 1844*, Marx begins from labor, from the ability of humans to consciously produce the conditions of their lives. While Marx’s conception of human nature does include transhistorical elements, a critique of alienation need not necessarily seek a return to some imagined and idealized past. This is because Marx’s view of human nature is both natural and social. Capitalism has separated humans from their ability to fulfill their biological and social needs by consciously and creatively acting in and on the natural world, while also creating new needs and new ways of producing. Thus, while the critique of alienation is grounded in reclaiming our species-being, this can only be realized through our *current* capacities for labor, the concrete level of development that human labor has attained.

The struggle against alienation, therefore, is a struggle over how we labor. It is, in other words, a struggle over how we spend our time. This is not, as Mau (2023) claims, simply a romantic appeal to a more authentic form of time. The struggle against alienation is not seeking a return to an untouched, pure natural state. It is an effort to exert control over the specific historical and social form that our ability to labor takes in the present. This would require control over the means of production and the ability to employ them in a way that responds to the needs of society, and this is only possible if we have control over our time. Mau is therefore only partially right when he states that “[t]he problem with abstract time is not that it is contrary to nature, but that it is a *means of oppression*” (242). Abstract time is indeed a means of oppression, but it is also contrary to our nature. It separates us from our essential human ability to consciously create the conditions of our lives and to fulfill our biological and social needs, subordinating this activity to the production of surplus-value. As Peter Hudis (2023) argues in the Introduction to Marx’s *Critique of the Gotha Program*, a disalienated society would be organized according to the *concrete* times of labor, the actual rhythms and pace of socially necessary tasks (9). While, as Marx (1990) states in *Capital*, labor-time in a communist society “serves as a measure of the part taken by each individual in the common labor, and of his share in the part of the total product destined for individual consumption” (172), this refers to the *concrete* activity and needs of the actually existing individuals that make up society, not the average time it would take an abstract individual to produce something or the abstract logic of surplus-value production for the market.

The point, therefore, is not to return to an imagined state of unmediated relations amongst humans and between humans and nature. As Mau (2023) rightly argues, there “is no such thing as a natural unity of humans and the rest of nature” (139). However, Mau assumes, incorrectly, that the political project of those who insist on the importance of alienation and the struggle against it is the end of *any* form of mediation. As Martin Hägglund (2021) writes in response to a similar critique of his views on alienation, “overcoming alienation does *not* mean overcoming objectification. For Marx, the need to objectify ourselves through material practices and social institutions is not in itself alienating.” Marx is not arguing for an end to objectification as such, which is simply the result of working on the material world; rather, he is trying to explain why, under capitalism, objectification has become alienating. The reason is that production under capitalism is mediated by the market, meaning concrete labor must be expressed as abstract labor. This process not only alienates workers from their labor, but it also transforms their time into the abstract time of value production.

It is certainly possible to articulate a theory of alienation and the struggle against it without incorporating the Romantic critique. Indeed, Hägglund (2020) defends a humanist Marx while rejecting the nostalgic impulse of Romanticism: “All such forms of nostalgia are misguided and ignore Marx’s fundamental insight that the commitment to social freedom for all became possible because of the historical advent of capitalism. There has never been a natural form of labor for spiritual beings and a primitive communism is neither possible nor desirable” (276). While Hägglund is right that there has never been a natural form of labor, there have been other, non-capitalist types that have been more aligned with the project of communist freedom. Hägglund’s view of democratic socialism is premised on increasing the realm of freedom by decreasing the amount of labor-time necessary to produce what is needed for a flourishing human life, “producing *for the sake of* increasing socially available free time rather than for the sake of generating profit” (277). While Hägglund rightly insists that “capitalism cannot be overcome through a linear continuation of the same mode of production, accompanied merely by a redistribution of wealth generated by proletarian labor” (276), he maintains that an increase in socially available free time can only be achieved through the development and implementation of technologies. While careful not to reproduce the Eurocentric vision of linear development, Hägglund nevertheless remains committed to a project of strictly forward-looking progress.^2^ As such, alternative paths toward communist freedom, including those expressed by anti-colonial resistance and indigenous lifeways, remain outside of his framework.

While both Mau and Hägglund are rightly critical of the reactionary tendencies that plague some of the Romantic tradition, Löwy and Sayre (2001) have shown that not all forms of Romanticism contain this reactionary impulse. The revolutionary Romantic orientation toward a selective recovery of elements of the past is wedded simultaneously to a forward-looking transformative vision for the future, and this does not need to rely on ideal representations of human-nature relations. Romantic critique need not be premised on the literal desire to return to an idealized past. Instead, it is a grounded recovery and forward-looking extension, a transformation of elements from moments in the past in which humans achieved a less alienated existence than in the present. As Vanessa Wills (2024) states, overcoming alienation is, in some ways, “a return—to life without economic classes and their attendant division of labor. And in other profound ways, it is something wholly new” (73). Rather than opposing an imagined utopian future to the material reality of the present, as the Utopian Socialists of Marx’s time did, Romantic Marxism opposes the actual, material potential of human society to the limits of the current one. Of course, Hägglund (2020) is correct that a return to past forms of living is neither possible—because these forms have themselves been transformed by the development of capitalism—nor always desirable. However, this does not mean that elements of the past cannot be included in present-day movements, as concrete examples of less alienated ways of life. Maintaining an ongoing relationship with a living history of non-capitalist lifeways and resistance allows us to remain open to possibilities that are often closed off to strictly forward-facing perspective. Indeed, the Romantic critique maintains a sense of hope in the possibility of realizing the potential of the past by combining past forms with anti-capitalist struggles in the present (Kompridis 2015).

Marx himself took extensive notes on non-Western societies, colonialism, and anti-colonial struggle later in his life (Anderson 2016, 2025). The greater focus on colonialism began to shift Marx’s views on anti-colonial resistance and struggles for national liberation. Kevin Anderson (2025) engages with Marx’s notes on the persistence of communal lifeways and resistance to colonialism as a way of showing Marx’s trajectory regarding the revolutionary potential of non-Western societies. Instead of either romanticizing pre-capitalist forms of living or viewing them as impediments to modern progress, Marx’s notes show a nuanced and dialectical understanding of pre-capitalist forms. Without idealizing the non-hierarchical and democratic nature of these pre-capitalist societies, Marx began to see the revolutionary potential of anti-colonial struggle. This is only possible because Marx saw the persistence of non-capitalist forms of life despite the tendency of capital to subordinate the concrete rhythms of human activity to the time of value production. Not merely an attempt to preserve or return to pre-capitalist forms of life, anti-colonial movements embody the struggle against capitalist alienation and the unilinear, Western path of modernity. Anti-colonial movements, therefore, represent particular expressions of the universal movement for human freedom.

Marx viewed resistance to colonialism in Latin America by indigenous communities as important and showed an admiration for the ways that communal forms of living were defended, despite the efforts of capital to subsume them to the law of value production (Anderson 2016). Similarly, his *Ethnological Notebooks* show that he was inspired by the more participatory democratic forms of Iroquois society, viewing it as a concrete example of an unalienated society in practice (Rosemont 2009). Furthermore, Marx saw the possibility of combining national liberation struggles in peripheral states with the communist movement in Western Europe. As Anderson (2025) has shown, Marx began to see Ireland as central to a communist revolution in England, if the Irish movement were to be combined with that occurring in England. And, in the 1882 Preface to the Russian edition of *The Communist Manifesto*, Marx and Engels (2002) argue that the communal *obshchina* in Russia could serve as “the point of departure for a communist development” (196) if combined with a proletarian revolution in Western Europe. As Wills (2024) argues, Marx saw the possibility of liberation in “echoes of the past” (163) that might be recovered as part of the struggle for a communist society.

Marx’s evolving views on colonialism and anti-colonial resistance are integrally connected to his romantic influence. Rather than viewing the past as closed, the relationship between spreading capitalist relations, colonial violence, anti-colonial resistance, and communal forms of living represents the possible recovery of concrete forms of life and struggle, as part of the movement toward a communist future. He did not express admiration for North American Indigenous communities, Irish anti-colonial resistance, or the Russian *obshchina* because he saw them as natural or unmediated ways of relating with the world. He admired them because he saw in them a more human way of relating with the world and because of the resistance these societies showed in the face of colonial violence. It is for this reason that Romantic Marxism insists on the ongoing dialectical relationship between the past, present, and future. The past contains the memory of concrete forms of living and struggling against the imposition of homogeneous abstract labor-time and, if combined with a global working-class movement in the present, could represent the basis of a future communist society. While Wills (2024) is right to distinguish Marx from both romantic anti-capitalism and Utopian Socialism, remaining open to the ongoing relationship with the past retains a sense of hope, not in an impossible return to a less alienated past, but in the material recovery of elements of that past as a way of realizing a communist future (Kompridis 2015). Building solidarity between indigenous and working-class movements, for example, can be seen as Romantic Marxism in practice, an effort to combine multiple concrete times in a struggle against the time of abstract labor. Indigenous resistance to the colonial imposition of capitalist relations—the opposition to the Line 9 pipeline by North American Indigenous communities, for example—defends still-existing traditional lifeways and points toward future, non-capitalist forms of living (Kipfer 2018). These forms of resistance seek to defend “not-just-capitalist” (486) ways of living and fight for other ways of life, other conceptions of time. Understood as a part of the universal struggle against capitalist alienation, anti-colonial resistance exemplifies the Romantic Marxist insistence that the possibility of a communist future emerges from the tragic-utopian dialectic of capitalist modernity. While the spread of capitalist relations has subordinated all forms of life to the logic of value and to the temporality of capitalist production, resistance to abstract labor-time persists in various ways. These moments of resistance retain a connection to the past which might be recovered and integrated as a part of a forward-looking communist movement intent on overcoming capitalist alienation in all its forms, thereby restoring our connection to our species-being, each other, and the natural world.

## Conclusion: “A Detour Through the Past”

Marx’s theory of alienation is not merely a misguided residue of his early humanist and romantic influence. He did not change his mind after 1844. Viewing Marx’s work as an evolutionary trajectory, this paper has traced the concept of alienation from the *Manuscripts of 1844*, through *Capital*, to his notes on pre-capitalist and indigenous societies from later in his life. The relationship between labor and needs runs through Marx’s theory and forms the basis of both alienation and commodity fetishism. By subordinating concrete human activity to the logic of value, capitalism tends to subsume all forms of human life to the time of capitalist production, measured as homogenous units of abstract labor-time. This process alienates workers from their essential human ability to fulfill their biological and social needs. However, despite this tendency, concrete forms of labor and time persist under capitalism. Retaining the humanist and Romantic elements of Marx’s work allows us to see the struggle against alienation as a struggle over time, an attempt to reclaim control over our ability to creatively act in and on the world. The Romantic appeal to past forms of life sees the possibility of selectively recovering elements of the past in order to apply them to our current moment. Romantic Marxism emerges from this contradictory relationship between tragedy and utopia, where an emancipatory future of communist freedom represents the realization of that which has been lost or destroyed by capitalist modernity, the possibility of an egalitarian, classless society.

But Romanticism is not a homogeneous tradition and its appeal to a sense of loss can take—indeed has taken—reactionary forms seeking a return to an imagined and idealized past. As Alberto Toscano (2010) shows in his work on fanaticism, Romantic appeals to the past have been used by reactionary forces to mobilize broad sentiments of discontent in a counter-revolutionary way, reviving aristocratic and racial-nationalist traditions. But the revolutionary current of Romanticism is directed toward an entirely distinct vision of the future through the selective recovery of elements of the past. It reclaims a liberatory future “via a *detour* through the past” (Sayre and Löwy 2021, 4). This is precisely the contribution that Romantic Marxism is capable of making, and it constitutes the link between Romanticism and Marx’s category of alienation properly understood. Like Marx, who viewed anti-colonial forms of resistance and communal traditions as a living, material history, the revolutionary current of Romanticism attempts to selectively recover elements from the past as part of a future-oriented, truly universal project. In contradistinction to reactionary nostalgia, Romantic Marxism views the past as a material, historical basis for political struggle seeking to end the alienation of human beings from our creative activity. This requires strategic, political intervention aimed at building solidarity between working-class and anti-colonial movements.

## Notes

1 For Marx (1990), socially necessary labor-time assumes the average time it takes to produce a commodity under the conditions of production of that society, which includes the average level of skill and intensity of labor. This means that a commodity produced by a lazy or incompetent worker would not contain more value than the same commodity produced in the average time: “Only labor-time expended under the average existing conditions of production *as well as* for the satisfaction of monetary social demand constitutes value” (Heinrich 2012, 51).

2 See Benjamin Noys (2014) for a related critique of Srnicek Nick and Williams, 2016 version of accelerationism. While, like Hägglund, Srnicek, and Williams argue that there are multiple possible trajectories that human development might take, they maintain that liberation can only occur through technological progress.
